# The Complex Interplay in Quantum Dot Neurotoxicity: From Environmental Exposure to Disruption of Neural Homeostasis

**DOI:** 10.3390/toxics14070558

**Published:** 2026-06-26

**Authors:** Haowei Xu, Faguang Kuang, Jiawei Yang, Qingzhong Wu, Yawen Du, Xiaosheng Tang, Baofei Sun

**Affiliations:** 1 Key Laboratory of Human Brain Bank for Functions, Diseases of Department of Education of Guizhou Province, College of Basic Medical, Guizhou Medical University, Guiyang 550025, China; 2 College of Optoelectronic Engineering, Chongqing University of Posts and Telecommunications, Chongqing 400065, China

**Keywords:** quantum dots, peripheral nervous system, central nervous system, toxicity mechanisms

## Abstract

Quantum dots (QDs) are semiconductor nanocrystals with unique photophysical properties, rendering them promising for applications in biomedical imaging, neuroscience, and various industrial sectors. However, the rapid expansion of their production and application inevitably leads to the release of QDs into the environment throughout their life cycle, classifying them as an emerging class of contaminants of concern. Their potential neurotoxicity not only represents a major bottleneck obstructing their clinical translation but also poses environmental and health risks that warrant serious attention. This review summarizes recent advances in the neurotoxicity of QDs, with a focus on their adverse effects on the central and peripheral nervous systems. It indicates that the mechanisms of QD neurotoxicity involve a complex network comprising oxidative stress, metabolic reprogramming, neuroinflammation, and multiple cell death pathways. Notably, the peripheral nervous system is highlighted as an early-warning target, and the significant risks associated with long-term, low-dose environmental exposure are emphasized.

## 1. Introduction

**Figure 1:**
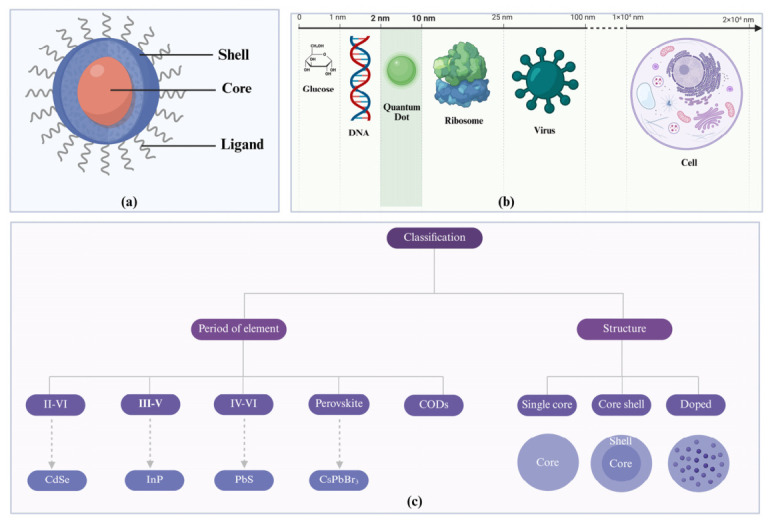
Basic structure, size range, and material classification of QDs. (**a**) Schematic diagram of a typical QD structure. The innermost core (e.g., CdSe, InP) is encapsulated by a shell (e.g., ZnS) to enhance photoluminescence and stability. Surface ligands confer colloidal solubility and enable further functionalization. (**b**) Size scale of representative nanoscale and biological entities, ranging from 1 nm to 2 × 10^4^ nm. Quantum dots (typically 2–10 nm in diameter) are larger than a glucose molecule (~1 nm) and DNA (~2 nm), but smaller than a ribosome (~25 nm), a virus (~100 nm), and cells (~10^4^–2 × 10^4^ nm). (**c**) Classification of QDs by elemental composition (II–VI, III–V, IV–VI), perovskites (e.g., CsPbBr_3_), and carbon dots (CDots). Structural variants include single-core, core/shell, and doped types. Representative materials such as CdSe, InP, PbS, and CsPbBr_3_ are listed.

The basic structure, size range, and material types of quantum dots are illustrated in Figure 1. A typical quantum dot features a three-layer core–shell–ligand architecture (Figure 1a): the core is a nanoscale semiconductor crystal, which is often coated with a shell material of a wider bandgap to passivate surface defects. The outermost layer consists of organic ligand molecules that stabilize dispersion, regulate solubility, and provide functionalization sites. Regarding size (Figure 1b), quantum dots typically have a diameter of 2–10 nm, placing them in a size range similar to biological entities such as glucose (~1 nm), DNA (~2 nm), ribosomes (~25 nm), and viruses (~100 nm). This similarity makes them suitable for bioimaging and cell labeling. Based on material composition and internal structure, quantum dots can be classified into groups such as II–VI, III–V, and IV–VI semiconductors, perovskites, and carbon quantum dots. They may also adopt single-core, core–shell, or doped structures (Figure 1c). Their unique quantum confinement effect endows them with exceptional optical properties [1,2]. These properties not only enable QDs to overcome the limitations of traditional dyes in biomedical applications such as biosensing, in vivo imaging, and disease diagnosis [3,4,5], but also facilitate their extensive use in industrial sectors, including Quantum Dot Light Emitting Diodes (QLED), solar cells, and catalysis [6,7,8]. However, this broad application potential is accompanied by exposure risks. During the production, use, and disposal of QDs, they may enter the natural environment through various pathways, including industrial wastewater, leachate from electronic waste, and atmospheric particulates [9,10,11,12]. Such exposure can induce toxicity in multiple organs and systems, leading to significant cytotoxicity, immunotoxicity, hepatotoxicity, and nephrotoxicity [13,14,15,16].

QDs, as nanomaterials, possess a small size and modifiable surface characteristics that enable them to traverse the blood–brain barrier, offering substantial advantages for the diagnosis and treatment of central nervous system (CNS) diseases. In recent years, QDs have facilitated non-invasive grading diagnoses of traumatic brain injuries, achieving sensitivity levels that reach the picomolar range [17]. They have also shown revolutionary potential in tracking the aggregation dynamics of α-synuclein (α-syn) in Parkinson’s disease, monitoring neurotransmitters, and developing brain–computer interfaces, all of which hold highly promising prospects for clinical translation [18,19,20]. However, this same characteristic raises concerns: environmental QDs may enter organisms via inhalation, drinking water, or the food chain [11,21,22,23], ultimately affecting the sensitive nervous system. Currently, the academic understanding of the neurotoxicity of QDs has evolved from the early simplistic model of “heavy metal ion release” to a more complex mechanism that encompasses “nanoscale effects.” Studies have demonstrated that QDs can induce ROS bursts, calcium signaling disorders, mitochondrial dysfunction, neuroinflammation activation, and even ferroptosis. These molecular events are intricately intertwined, forming a complex toxicity network that ultimately leads to neuronal death, impaired synaptic plasticity, and behavioral deficits. Consequently, research on the neurotoxicity of QDs has transcended the mere realm of “clinical translation” and has become a critical issue of “environmental safety”.

**Figure 2:**
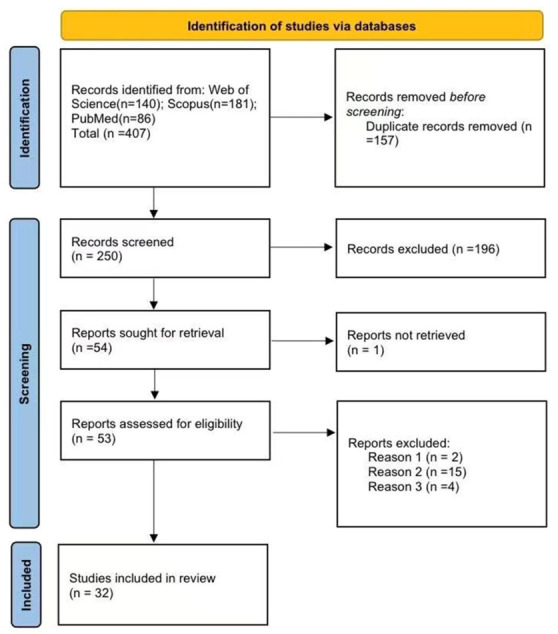
Flow diagram of the literature screening process. Initial database searches yielded 407 records, and 157 duplicates were removed. After title and abstract screening, 53 records were selected for full-text assessment. During the full-text assessment, 21 records were excluded for the following reasons: primary outcome measures unrelated to the nervous system (*n* = 2), insufficient methodological details to interpret neurotoxicity results (*n* = 15), and commentary articles lacking original data (*n* = 4). Ultimately, 32 studies were included for discussion.

This review summarizes the current state of research on the neurotoxicity of QDs. First, we briefly introduce the fundamental properties of QDs and their rapid development and applications in biomedicine. Subsequently, through literature retrieval and screening (Figure 2), we focus on the toxic effects of QDs on both the peripheral and central nervous systems. On this basis, we further explore the multilayered mechanisms underlying QD toxicity and summarize the currently effective mitigation strategies. Finally, we analyze the limitations and challenges of existing studies and provide a preliminary outlook on potential future research directions. Through this synthesis, we aim to help readers better understand the current landscape of QD neurotoxicity.

## 2. Properties and Biological Applications of QDs

### 2.1. Characteristics of QDs

QDs, as typical semiconductor nanocrystalline materials, derive their properties fundamentally from pronounced quantum confinement effects at the nanoscale [24]. when at least one dimension of a material approaches or falls below the exciton Bohr radius, charge carriers become strongly confined spatially, inducing a transformation of the band structure from continuous to discrete, atom-like energy levels [25,26]. This quantized reconstruction of the electronic structure serves as the physical basis for understanding the unique properties of QDs. The lower-left and upper-right property panels in Figure 2 intuitively summarize the resulting key properties, which will be elaborated in detail in the following text.

In terms of optical characteristics, QDs exhibit size-dependent emission behavior [27]. Due to the quantum confinement effect, their bandgap energy exhibits a negative correlation with crystal size, enabling continuous tuning of the emission wavelength across the entire visible to infrared spectrum through precise control of synthesis process parameters [28]. Furthermore, QDs feature broad-spectrum absorption and narrow peak emission. Combined with a high fluorescence quantum yield (up to 90% or more) and excellent photostability, QDs demonstrate significant advantages over traditional organic dyes in multicolor imaging and display technologies [7,29]. Additionally, the significant Stokes shift effectively avoids crosstalk between excitation light and emission signals, providing ideal conditions for high signal-to-noise ratio detection. These optical properties (size tunability, high quantum yield, broad absorption, narrow emission, and photostability) form the basis of the bioimaging and molecular detection applications shown in Figure 2.

Regarding electrical properties, QDs exhibit efficient charge separation and transport capabilities [30]. Their nanoscale dimensions allow photogenerated carriers to rapidly migrate to the surface for redox reactions, while tunable energy levels facilitate the design of functional heterojunction structures. Notably, the surface states of QDs have a decisive impact on their optoelectronic properties. Surface ligands not only maintain colloidal stability through steric hindrance effects but also serve as regulatory channels for energy and charge transfer [31]. Through ligand engineering, efficient coupling of QDs with biomolecules, polymer matrices, or electrode materials can be achieved, laying the foundation for their applications in interdisciplinary fields. These properties underpin the applications shown in Figure 2, such as drug delivery and adjuvant cancer therapy.

Based on these characteristics, QDs have achieved revolutionary applications in multiple cutting-edge technological fields. Although significant progress has been made in quantum dot technology, its future development still faces important challenges. First, the biocompatibility and potential environmental risks of heavy metal-containing QDs require urgent attention, prompting exploration of alternative material systems including silicon QDs, carbon QDs, and lead-free perovskite QDs [32,33,34]. Second, enhancing the long-term stability of materials is crucial for realizing their industrial applications.

### 2.2. Applications of QDs in Brain Diseases

**Figure 3:**
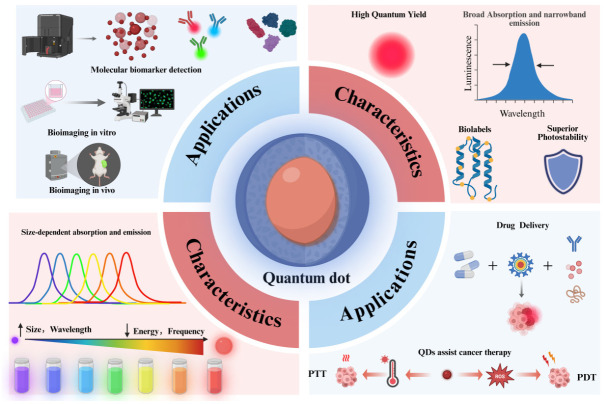
Schematic overview of quantum dot (QD) properties and their multifaceted biomedical applications. QDs exhibit size-tunable emission wavelengths, high quantum yield, broad absorption spectra, narrow emission bands, and excellent photostability (property panels: **lower left** and **upper right**). These properties enable diverse applications, including bioimaging, molecular detection, drug delivery, and adjuvant cancer therapy (application modules: **upper left** and **lower right**).

Owing to their unique fluorescence properties and excellent biocompatibility, QDs hold significant promise for integrated diagnosis and therapy of major brain diseases. Figure 3 summarizes the multifaceted biomedical applications of quantum dots based on their core properties. In the context of disease diagnosis, electrochemical sensors based on QDs enable sensitive detection of neurotransmitters, thereby offering new strategies for early disease screening [35]. QDs probes facilitate the ultra-sensitive identification of glioma cells [36,37]. Furthermore, near-infrared II QDs achieve high-resolution, high signal-to-noise ratio imaging of deep brain tissues, effectively overcoming the limitations of traditional imaging techniques regarding penetration depth and spatial resolution [38]. In a traumatic brain injury model, novel gallium-doped copper indium selenide QDs enhance near-infrared fluorescence performance, thereby supporting comprehensive monitoring from preoperative localization to intraoperative navigation and postoperative evaluation [17,39]. Additionally, in research on neurodegenerative diseases such as Alzheimer’s and Parkinson’s, QDs act as molecular probes for real-time and dynamic tracking of the aggregation processes of pathological proteins like Aβ and α-syn, providing essential visualization tools for elucidating the molecular mechanisms underlying disease onset and progression [40,41,42,43].

In the therapeutic domain, QDs exhibit significant advantages as well. In brain tumor therapy, QDs leverage their nanoscale properties to serve as drug carriers that efficiently cross the BBB, enabling targeted drug delivery and controlled release to the lesion area. This strategy not only enhances local drug concentration but also significantly reduces systemic toxicity [44]. Moreover, quantum dot-mediated photodynamic therapy (PDT) and photothermal therapies (PTT), in combination with chemotherapy, have demonstrated considerable synergistic tumor-suppressing effects, offering new insights for enhancing antitumor efficacy [45]. For neurodegenerative diseases, QDs present a novel approach to multimodal treatment of Parkinson’s disease by mitigating oxidative stress, facilitating synergistic drug delivery, inhibiting the fibrillation of α-syn, and disaggregating preformed fibrils [46,47]. Functionalized QDs show potential for multitarget intervention in Alzheimer’s disease by simultaneously inhibiting Aβ oligomerization and tau protein hyperphosphorylation, scavenging ROS, alleviating neuroinflammation, and synergistically maintaining mitochondrial membrane potential and calcium homeostasis [48,49,50,51].

In summary, quantum dot technology integrates diagnostic and therapeutic functions cohesively in the field of brain diseases. At the diagnostic level, it provides multi-scale, high-precision imaging solutions that range from cellular levels to vascular networks; at the therapeutic level, it opens new avenues for enhancing treatment efficacy through various mechanisms such as targeted delivery, synergistic enhancement, and neuroprotection. With further optimization of biosafety, QDs are poised to become a pivotal platform technology driving advancements in precision medicine for neurological diseases.

## 3. Neurotoxicity of Quantum Dot

QDs exhibit considerable potential in the biomedical sector, particularly in the field of neuroscience. Their nanoscale dimensions and modifiable characteristics enable them to traverse the blood–brain barrier (BBB), providing a significant advantage for the diagnosis and treatment of CNS disorders. However, the very properties that enhance the applicability of QDs may also contribute to their neurotoxicity. Although the BBB is designed to protect the nervous system [52], the nanoscale size and surface modifications of QDs can exploit the nutrient transport channels of the BBB or gain entry into the CNS through alternative pathways [53], such as the olfactory nerve pathway [54]. This can result in selective accumulation of QDs in brain tissue, potentially leading to localized damage. Furthermore, the toxicity of QDs is highly contingent upon their physicochemical properties, including size, surface charge, concentration, surface modifications (such as ligands and coatings), core composition, solubility (and degradation behavior), and purity [55,56,57]. Compared to other organ systems, damage to the nervous system is often irreversible and severe, with toxic responses potentially occurring at lower doses or within shorter time frames [58,59]. Table 1 and Table 2 provide summaries of the toxicity of QDs to both the central and peripheral nervous systems. (PNS).

### 3.1. The CNS Effects of QDs

The CNS serves as the primary structure for integrating neural information and executing complex functions [91]. However, it is often subject to irreversible neuronal loss following injury, which leads to a range of severe neurological deficits, including sensory-motor dysfunction, cognitive decline, emotional disorders, and epilepsy [92,93]. Research has shown that the direct pathological basis of several severe neurodegenerative diseases, such as Parkinson’s disease and Alzheimer’s disease, is the extensive damage and death of neurons [94,95]. Given the challenges associated with repairing CNS injuries, the exploration of their mechanisms and protective strategies remains a prominent focus within neuroscience [96]. Additionally, the CNS is a potential critical target organ for nanoparticles (NPs) [97]. QDs, as a representative type of nanomaterial, can penetrate the CNS through various pathways, including crossing or disrupting the BBB [98,99], directly entering brain tissue via the nose-to-brain pathway [54], and permeating through channels associated with cerebrospinal fluid (CSF) circulation [53]. Once inside the CNS, QDs can induce a range of neurotoxic effects, such as neuronal apoptosis, abnormal activation of glial cells, synaptic dysfunction, and disruptions in neurotransmitter homeostasis, among other pathological alterations [100,101,102].

However, a comparative analysis of the studies listed in Table 1 reveals that, although the neurotoxicity of QDs has been confirmed across multiple models, differences in experimental models, exposure doses, and QD compositions among these studies directly affect the generalizability of toxicity patterns and the reliability of risk assessment. From the existing literature, two relatively clear trends can be identified. First, the toxic dose threshold for direct brain entry routes (e.g., intranasal instillation, intravenous injection) is significantly lower than that for peripheral routes (e.g., oral gavage, intraperitoneal injection). Intranasal instillation of carbon-based QDs at 0.5–5 mg/kg for several days to 28 days induces damage in brain regions such as the olfactory bulb and hippocampus [23,76]. Intravenous injection of CdSe/ZnS QDs at as low as 0.5 nM suppresses hippocampal synaptic plasticity [75]. In contrast, oral gavage at 10–40 mg/kg/day for 30 consecutive days is required to produce obvious behavioral abnormalities [86]. These findings indicate that the risk threshold for direct brain exposure is approximately 1–2 orders of magnitude lower than that for peripheral exposure, and thus exposure route must be considered when assessing risk. Second, the sensitivity of different biological models follows a rough hierarchy: nematodes are the most sensitive (toxic at 10–100 μg/L) [60,82], followed by mice (mg/kg range) [69,85], then primary neurons, with immortalized cell lines showing the highest thresholds but consistent trends [67,81]. This gradient provides a basis for multi-model integration, although quantitative conversion factors are currently lacking.

Based on these findings, several unresolved issues merit attention. First, non-monotonic dose–response relationships have been observed. For example, graphene oxide quantum dots (GOQDs) induced the strongest anxiety-like behavior in zebrafish at the lowest tested concentration (12.5 µg/mL) [84]. For CdTe QDs, smaller particles were more toxic at low to medium concentrations, but this trend reversed at high concentrations [68]. This suggests that toxicity mechanisms may shift across dose ranges. Future studies should cover a full range from no effect to a plateau or decline in effect during dose-finding phases to identify non-monotonic intervals. Second, data on chronic low-dose exposure are almost entirely absent, with most studies being acute or subacute (days to four weeks) [23]. Given that QDs may persist in the brain for long periods, long-term low-dose exposure experiments lasting 3–6 months are recommended to observe behavioral and neuropathological changes over extended timeframes. Finally, differences in animal strains, solvent media, dosing frequency, and detection time windows across studies further complicate cross-literature comparisons.

In summary, current evidence establishes two main patterns: the threshold for direct brain exposure is lower than that for peripheral routes, and model sensitivity follows a qualitative gradient. Non-monotonic responses, lack of anchoring for extrapolation, and missing chronic exposure data are major methodological bottlenecks. Future research should prioritize quantitative cross-model comparisons, complete dose range coverage, and long-term exposure assessments to move the neurotoxicity evaluation of QDs toward quantitative prediction.

### 3.2. The Toxic Effects of QDs on the PNS

The PNS comprises cranial nerves, spinal nerves, and their associated ganglia, functioning to connect the CNS with various organs throughout the body and to transmit sensory and motor signals [103]. In contrast to the CNS, the extracellular environment of the PNS is more open and lacks a stringent protective structure akin to the BBB, rendering the PNS more susceptible to exposure to NPs in the circulatory system. Research has demonstrated that inhaled QDs can penetrate the PNS via the olfactory nerve [54]. Notably, the PNS exhibits a stronger intrinsic regenerative capacity than the CNS, primarily due to the ability of Schwann Cells to dedifferentiate into a repair phenotype following injury, secrete neurotrophic factors, and form Büngner bands, which provide guidance and support for axonal regeneration [104,105]. However, exposure to QDs may severely disrupt this repair process. It is particularly important to emphasize the close structural and functional interconnection between the PNS and CNS. Quantum dot-induced peripheral nerve damage not only results in local functional impairment but may also indirectly disrupt the homeostasis of the CNS by diminishing the delivery of neurotrophic factors, triggering neurogenic inflammation, and causing abnormal signal transduction, thereby advancing the pathological progression of clinical diseases such as Alzheimer’s disease [106,107].

The toxicity of CdTe QDs to peripheral nerve cells may be greater than that of central neurons. Early studies by Manasi P Jain et al. indicated that the toxicity of CdTe QDs to PNS models increased with system complexity, with primary DRG cultures displaying higher sensitivity than the PC12 cell line [87]. However, it is essential to note that the PC12 cell line, derived from rat adrenal pheochromocytoma, exhibits significant metabolic, antioxidant defense, DNA repair, and apoptosis pathway differences compared to primary normal neurons. Moreover, tumor cell lines are generally more resistant to toxic substances. To further compare the toxicity between peripheral and central neurons, it is necessary to investigate the effects of CdTe QDs on both primary DRG neurons and primary cortical neurons. Furthermore, researchers discovered that the antioxidant lipoic acid (LA) paradoxically enhanced toxicity in the DRG model, suggesting that the surface chemistry of QDs might influence the toxicity mechanism by regulating cellular internalization efficiency. This contradictory mechanism requires urgent elucidation. The study by Changcun Bai’s team on peripheral neurotoxicity observed that CdTe QDs induced significant toxic effects on both ND7/23 and RSC96 cells [88,89,90]. The survival rates of both cell types exhibited a dose-dependent decrease, while the apoptosis rates significantly increased. Ultrastructural analysis revealed morphological changes in the cells, including the disappearance of mitochondrial cristae, swelling, and endoplasmic reticulum expansion and hyperplasia. The intracellular levels of ROS) were significantly elevated, and the mitochondrial membrane potential (ΔΨm) markedly decreased. The formation of numerous autophagic vacuoles was also observed in RSC96 cells. Notably, the zebrafish developmental model demonstrated that exposure to ultra-low doses (≥100 nM) was sufficient to inhibit motor neuron axon growth, leading to a sharp decrease in swimming speed and reduced activity time. At exposure levels close to environmentally relevant concentrations, QDs were sufficient to cause significant disturbances in the development of the nervous system in aquatic organisms, which is directly related to the health of aquatic ecosystems [108].

Research in this field is still in its infancy, highlighting an urgent need to integrate nanotoxicology with clinical neurology methods. This integration aims to elucidate several core issues: Whether the long-term retention of quantum dots disrupts the inherent regenerative capacity of the peripheral nervous system, and the concurrent establishment of a panel of peripheral nervous system-specific biomarkers.; and the concurrent establishment of a PNS-specific biomarker panel. This panel may include indicators such as abnormal fluctuations in lipid droplets and a reduction in neurite growth rate, which can help define quantitative parameters for establishing biological safety thresholds in nanocarrier design. A systematic evaluation of the peripheral neurotoxicity of QDs is essential, not only as a necessary complement to their central toxicity but also as a proactive measure to warn against overall neurological risk, starting from the more exposed ‘periphery.’ This approach will advance the critical scientific foundation for the safe translation of QDs in future applications.

## 4. Toxicity Mechanisms and Attenuation Strategies

### 4.1. The Mechanism of Neurotoxicity of QDs

**Figure 4:**
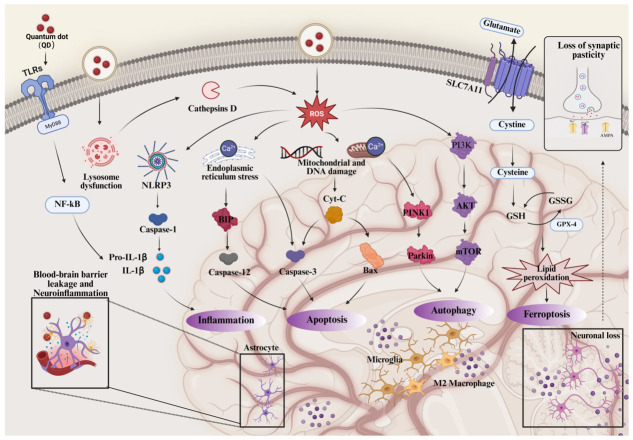
Proposed mechanisms of QD-induced neurotoxicity include neuroinflammation, organelle dysfunction, and activation of apoptosis, ferroptosis, and autophagy, collectively leading to neuronal loss and impaired synaptic plasticity.

The neurotoxicity of QDs manifests as a multi-level, interconnected dynamic network process. This process begins with oxidative stress and ion homeostasis imbalance, undergoes cascading amplification via neuroinflammatory responses, and ultimately leads to neuronal death and functional impairment. Figure 4 summarizes the three core components of this toxicity network—oxidative stress/calcium dysregulation, neuroinflammation, and multiple cell death pathways—as well as their interrelationships, thereby providing a visual framework for the subsequent detailed discussion of mechanisms at each level.

#### 4.1.1. Oxidative Stress-Calcium Homeostasis Imbalance

ROS are a group of highly reactive oxygen-containing molecules generated during aerobic metabolism in cells, which play significant signaling roles [109,110]. Excessive ROS can induce lipid peroxidation of bio membranes, nucleic acid breakage, and protein denaturation, leading to irreversible cellular damage. These processes are closely associated with the onset of neurodegenerative diseases such as Parkinson’s disease and Alzheimer’s disease [111,112]. Upon entering neural cells, QDs trigger two critical initial events through surface chemical reactions and interactions with organelles: a burst of ROS generation accompanied by DNA damage [60,65], and disruption of intracellular calcium ion ([Ca^2+^]i) homeostasis [62,63,79]. Current studies have confirmed a positive feedback loop between these two events, forming the core of a toxic network. Specifically, ROS directly induces oxidative damage and activates stress pathways, such as MAPK and NF-κB [60,66]. Mitochondria-derived ROS (mtROS) also activates the nuclear factor erythroid 2-related factor 2 (Nrf2)/PTEN-induced kinase 1 (PINK1)-mediated mitophagy process [80]. Conversely, calcium overload promotes the generation of more ROS by disrupting mitochondrial function [90,113]. Notably, quantum dot-induced calcium overload exhibits a unique nanoscale effect; their surface properties can interfere directly with the function of voltage-gated sodium channels (VGSCs), triggering persistent sodium influx. This, in turn, leads to secondary calcium overload through VGCCs and the reverse sodium-calcium exchanger (NCX) [62,63]. This positive feedback mechanism not only directly damages biomacromolecules but also provides a continuous driving signal for the activation of the entire toxicity network.

However, existing evidence primarily derives from high-dose acute exposure models, and the ability to trigger and maintain this positive feedback loop under environmentally relevant low-dose long-term exposure remains unclear, directly affecting the accuracy of health risk assessments. Furthermore, the antioxidant N-acetylcysteine (NAC) can only partially alleviate cell death [68]. Once this core engine is activated, its downstream cascade reactions may gain autonomy, making it challenging to completely block through intervention strategies targeting a single point.

#### 4.1.2. Metabolic Reprogramming and Persistent Neuroinflammation

Upon recognizing the initial stress signals from microglia, the amplification phase of the toxic network is initiated. At the level of pattern recognition receptors, the surface characteristics of QDs and the ROS they induce can be recognized by TLR2, which subsequently activates the downstream TLR2/MyD88/NF-κB signaling transduction pathway. This pathway promotes the transcription of multiple pro-inflammatory factor genes, including pro-IL-1β [67]. Concurrently, the endocytosis of QDs disrupts lysosomal membrane stability, triggering the assembly of the NLRP3 inflammasome and leading to the maturation and secretion of IL-1β through the caspase-1-dependent pathway [60,67,70,77]. Furthermore, studies have confirmed that QDs can activate various immune-related pathways, including the NOD-like receptor signaling pathway and the tumor necrosis factor signaling pathway, thereby forming a complex immune response network.

Importantly, recent research has highlighted the significant role of metabolic disorders in driving neuroinflammation. Exposure to QDs can induce widespread metabolic disturbances that affect fundamental metabolic pathways such as glucose metabolism, lipid metabolism, and amino acid metabolism [72]. Additionally, it specifically interferes with key neural metabolic processes, including the arginine-proline metabolic axis, unsaturated fatty acid biosynthesis, and the glutamine-glutamate metabolic cycle, thereby exacerbating neuroinflammatory responses [76]. Notably, quantum dot exposure can induce a shift in microglial energy metabolism from oxidative phosphorylation to aerobic glycolysis. This metabolic phenotype switch is regulated by the mTOR signaling pathway, which provides the necessary energy and metabolic precursors for the polarization of microglia towards the pro-inflammatory M1 phenotype [69,72]. These findings indicate that QDs not only act as exogenous danger signals to activate immune responses but also reconstruct intracellular metabolic states, providing a metabolic foundation for maintaining persistent inflammatory phenotypes. Under conditions of sustained immune activation, activated microglia release significant amounts of inflammatory mediators, including TNF-α and IL-1β, which further exacerbate neuronal oxidative stress and mitochondrial dysfunction, thereby forming a self-sustaining positive feedback loop of inflammation and oxidative stress, significantly amplifying the initial damage effects.

Although research on microglial polarization and metabolic reprogramming has made progress, the regulatory network underlying quantum dot neurotoxicity still requires systematic elucidation. Current studies primarily focus on the pro-inflammatory M1 phenotype, while the regulatory mechanisms of the repair-promoting M2 phenotype and the dynamic balance between these two phenotypes during quantum dot exposure remain poorly understood. It is noteworthy that QDs can induce immune suppression [114] or trigger intense inflammatory responses [60,67] under different experimental conditions, suggesting that their immunomodulatory effects are significantly context-dependent. However, the key regulatory factors determining this biphasic response, including the dose-effect relationship, the specific influence of surface chemical properties, and the kinetics of exposure time, remain unclear, reflecting the complex nature of the interaction between QDs and the neuroimmune system.

#### 4.1.3. Diverse Neuronal Death and Dysfunction

Under the synergistic effects of oxidative stress and inflammatory factors, neurons experience death or dysfunction through multiple parallel and interacting signaling pathways, representing the ultimate execution terminal of the quantum dot neurotoxicity network. In the context of programmed cell death pathways, QDs can activate the classical mitochondrial apoptosis pathway by upregulating the pro-apoptotic protein Bax and inhibiting the anti-apoptotic protein Bcl-2. This induced mitochondrial dysfunction leads to membrane potential collapse, cytochrome c release, and ultimately activates caspase-3 to execute the cell death program [68,89]. Additionally, endoplasmic reticulum stress serves as another critical pathway that can induce apoptosis via the caspase-12-caspase-7-PARP cascade reaction. Furthermore, ferroptosis, a regulated cell death modality characterized by iron dependence and abnormal accumulation of lipid peroxides, is recognized as a universal mechanism of nanomaterial-induced neurotoxicity. Studies have shown that QDs can specifically activate this pathway by downregulating the expression of the key antioxidant enzyme GPX4 [23]. Recent evidence further reveals that CdSe/ZnS QDs can interfere with molecular pathways of neural development through the “ferroptosis-TCA cycle axis.” Notably, upstream disturbances in calcium signaling may exacerbate abnormal intracellular iron accumulation through the “calcium-iron crosstalk” mechanism, vividly illustrating the complex interactions among various hierarchical signaling pathways within the toxicity network.

Before the onset of cell death, progressive damage to subcellular structures and functions has already begun, providing a key mechanism to explain cognitive impairment under low-dose exposure. QDs promote abnormal autophagy flux through oxidative stress, resulting in excessive degradation of key presynaptic functional proteins such as Synapsin-I, which directly impair synaptic plasticity [61]. Simultaneously, they inhibit ERK activity by upregulating the expression of DUSP2 and PTPN7 genes, while synergistically downregulating PI3K-Akt pathway signaling, ultimately leading to reduced expression of the transcription factor c-Fos, which is closely associated with memory formation [66]. Particularly noteworthy is the regulation of the cAMP-CREB-BDNF signaling axis, which is central to neuronal survival and synaptic plasticity, by QDs, further exacerbating the imbalance in cellular activity and functionality [74]. These findings mechanistically explain a significant phenomenon: under environmentally relevant low-dose exposure, cognitive impairments such as learning and memory deficits can manifest even in the absence of substantial neuronal loss, indicating that synaptic dysfunction serves as an earlier and more sensitive endpoint of neurotoxicity than cell death.

Although current research has identified various terminal phenotypes of neuronal death and dysfunction induced by QDs, the intrinsic regulatory hubs and decision-making mechanisms remain largely unelucidated. Our current understanding is significantly limited regarding the determinants that dominate cell death pathways under specific exposure conditions, as well as the relative contributions of QDs’ physicochemical properties and the intrinsic metabolic state of target cells. This ambiguity at the mechanistic level severely constrains the development of precise neuroprotective strategies targeting cognitive deficits induced by QDs.

#### 4.1.4. Structure–Activity Relationships in QD Neurotoxicity

In neurotoxicity studies of QDs, the relationship between structural parameters and toxic phenotypes is becoming clearer through cross-material comparisons. Particle size is a key factor determining brain distribution and subcellular damage. Ultra-small QDs (<5 nm) can more easily cross the blood–brain barrier or enter the brain parenchyma via the olfactory nerve pathway. For example, carbon quantum dots (CQDs) with a core size of ~2.95 nm accumulate in the olfactory bulb and hippocampus after intranasal exposure [76]. Silicon quantum dots (SiQDs) as small as 1.44 nm induce dopaminergic neuron loss at very low concentrations [82]. However, smaller size does not always result in higher toxicity. In the same mercaptopropionic acid (MPA)-capped CdTe QD system, 3.5 nm particles induce more extensive transcriptomic perturbations and severe inflammatory infiltration after local hippocampal injection than 2.2 nm particles. This suggests that larger particles may cause stronger chronic immune stimulation due to their in vivo retention kinetics [66,71].

Surface chemistry also significantly influences toxicity pathways. CQDs rich in oxygen-containing functional groups (e.g., hydroxyl and carbonyl) are readily internalized by cells and retained in lysosomes, damaging lysosomal membranes and triggering multiple programmed cell death pathways [76,85]. Nitrogen-doped or amino-functionalized graphene quantum dots (GQDs) induce microglial circRNA reprogramming and enrich inflammatory pathways such as TNF/NF-κB. In contrast, GOQDs primarily cause neurobehavioral abnormalities by disrupting the kynurenine metabolic pathway [83,84]. Although negative surface charge contributes to dispersion stability, it does not ensure safety. MPA-capped CdTe QDs (ζ ≈ −22.5 mV) cause DNA damage, whereas polymer-coated CdSe/ZnS QDs, stable at neutral pH, release Zn^2+^/Cd^2+^ in the acidic lysosomal environment, leading to sublethal proliferation inhibition [64,65].

Core composition and shell structure further determine bias in toxicity pathways. Cadmium-containing QDs (e.g., CdTe, CdSe) generally rely on Cd^2+^ release and ROS generation to activate the NLRP3 inflammasome. Coating with a ZnS shell partially alleviates toxicity but does not fully eliminate microglial metabolic reprogramming and M1 polarization [69,70,72]. In contrast, carbon-based QDs do not rely on metal ion release; they drive neuroinflammation mainly by disrupting arginine/proline metabolism and unsaturated fatty acid synthesis [76]. Silicon QDs act through the autophagy–ferroptosis axis, and this effect is reversed by iron chelators [82]. CsPbBr_3_ perovskite nanoparticles cause developmental toxicity via Pb^2+^ release; coating with a SiO_2_ shell significantly reduces lead accumulation and alleviates toxicity [78,79].

Although the above comparisons reveal several structure–activity trends, methodological limitations and contradictory conclusions in current research must be acknowledged. First, most studies lack systematic gradient controls of particle size, surface charge, or core composition within the same experimental system. Structure–activity conclusions are often drawn from indirect cross-literature comparisons rather than strictly controlled variable experiments. Second, even the same material system can show opposite size-toxicity trends under different exposure models, highlighting that structure–activity relationships are far more complex than simple linear extrapolation. In primary neurons in vitro, 2.2 nm MPA-capped CdTe QDs induce higher acute apoptosis than 3.5 nm particles. However, after hippocampal injection in vivo, the 3.5 nm particles cause broader transcriptomic perturbations and greater inflammatory infiltration [68,71]. This contradiction suggests that in vivo clearance kinetics and local retention may reverse toxicity predictions based on in vitro specific surface area; structure–activity relationships must be interpreted in the context of complete biodistribution. Finally, comparisons of core composition and shell structure lack proper toxicity benchmarks. Although the CdTe versus CdTe@ZnS pairing demonstrates the attenuating effect of the ZnS shell, the shell itself is not inert—Zn^2+^ released under acidic lysosomal conditions also exhibits neuroactivity [64]. More critically, no study has systematically compared cadmium-based QDs with low-toxicity alternative materials (e.g., InP/ZnS, Ag_2_Se, carbon dots) under identical conditions. Thus, the fundamental structure–activity question of which core composition is safer remains unresolved.

In summary, current structure–activity analyses of quantum dot neurotoxicity have accumulated valuable but fragmented evidence. However, due to the lack of systematic gradient controls, inconsistencies between in vivo and in vitro models, and neglect of dynamic biological barriers and protein corona effects, there remains a considerable gap toward establishing quantitative predictive models. Future research should prioritize synthesizing libraries of QDs with orthogonal variations in size, charge, and shell structure under unified platforms. These should be combined with real-time in vivo distribution monitoring and multi-omics endpoints. Moreover, low-toxicity alternative cores should be included as benchmark controls under identical exposure conditions. Only then can structure–activity relationships truly serve the safety-by-design of nanomaterials.

### 4.2. Potential Pathways to Reduce the Neurotoxic Effects of QDs

In recent years, the in-depth exploration of the neurotoxicity mechanisms of QDs has prompted researchers to actively investigate various intervention strategies aimed at mitigating their neurotoxic effects and facilitating their clinical translation. These strategies emerge from three major dimensions: material engineering optimization, pharmacological intervention, and exposure control. Firstly, the development of heavy metal-free QDs has become a prevailing trend [115,116]. Studies have shown that CQDs without surface functionalization or cell membrane coating can still induce neuroinflammation, oxidative stress, and neuronal damage, exhibiting certain neurotoxicity [23,76,85]. However, a recent study proposed a strategy: the researchers prepared caffeic acid-conjugated carbon quantum dots and encapsulated them with microglial cell membranes to construct a biomimetic nanovesicle (CDs-CA-MGs). Administered intranasally to bypass the blood–brain barrier, these nanovesicles achieved homologous targeting of neuroinflammatory sites and significantly suppressed neuroinflammation while improving cognitive function in a mouse model of Alzheimer’s disease [117]. This finding suggests that cell membrane encapsulation not only enables precise delivery but may also mask or reduce the potential toxicity of the carbon quantum dot core. Building on this, other homologous targeting surface modification methods, such as CD47 protein modification, warrant further exploration. Nevertheless, it should be recognized that although CDs-CA-MGs have shown favorable short-term efficacy, the study did not systematically evaluate their long-term biosafety—particularly whether microglial cell membranes, as xenogeneic biomaterials, may induce immunogenic responses (e.g., anti-membrane antibody production or complement activation) after repeated administration, and whether they alter the in vivo distribution and metabolic clearance of carbon quantum dots. These critical questions remain to be addressed. Furthermore, while cell membrane coating strategies achieve “stealth” and targeting functions, they may also introduce unpredictable biological effects due to the complexity of membrane proteins, presenting challenges for clinical translation in terms of immunogenicity and batch-to-batch stability.

Second, for conventional cadmium-based quantum dots, the construction of a core–shell structure can effectively encapsulate the toxic core and significantly reduce the leakage of heavy metal ions [14]. However, the core–shell structure is not completely sealed; the shell may undergo corrosion or degradation during long-term in vivo circulation, leading to slow release of the toxic core. In addition, surface functionalization is critically important. Modifications such as PEGylation or conjugation with natural antioxidants (e.g., α-lipoic acid) can enhance biocompatibility, reduce non-specific interactions with biomolecules, and confer additional antioxidant or chelating functions [118,119]. Although these strategies are effective, it should be critically recognized that surface modification itself may introduce new toxicity risks. Notably, studies have found that even commonly used PEGylated quantum dots can significantly inhibit neurite outgrowth [120], highlighting that the density, chain length, and charge distribution of modifying molecules may exert unpredictable effects on neural cells. Systematic assessments of such “coating-induced toxicity” remain lacking.

Finally, targeting well-defined toxicity pathways with specific inhibitors or protective agents represents another effective strategy. However, such pharmacological interventions often alleviate only some downstream toxic effects and are unlikely to fundamentally resolve the issue of heavy metal ion leakage. Moreover, the neurotoxicity of the inhibitors themselves requires rigorous validation. The development of degradable quantum dots could help reduce their accumulation in the nervous system and mitigate long-term toxicity. Nevertheless, the composition and toxicity of degradation products, as well as precise control over degradation rates, remain challenging problems that urgently need to be addressed.

## 5. Summary and Outlook

QDs, as nanomaterials with unique photophysical properties, have demonstrated significant potential for applications in the biomedical field, particularly in the diagnosis and treatment of neurological diseases. However, their complex mechanisms of biological toxicity, especially the potential harm to the nervous system, present a critical bottleneck that limits their clinical translation. Although current research has amassed a substantial body of toxicity phenotype data, our understanding of the underlying mechanisms remains incomplete, and discrepancies among research findings persist. Notably, the same type of QDs exhibits varying toxicity thresholds across different model systems, underscoring the urgent need for a standardized evaluation system. To ensure the safe application of QDs, it is essential to achieve breakthrough progress in material design, toxicity mechanism research, and safety evaluation.

First and foremost, the material design philosophy of “safety first” should be prioritized. This can be accomplished by developing novel low-toxicity QDs and optimizing surface modification strategies to minimize their biological toxicity at the source. It is noteworthy that current research on cadmium-free QDs remains in its infancy, with their long-term toxicity and metabolic patterns yet to be fully elucidated. While surface modification strategies enhance biocompatibility, they may inadvertently alter the biodistribution and barrier-penetrating capabilities of QDs. This effect is particularly critical yet often overlooked in the context of the nervous system. Future research should intensify the investigation of BBB penetration mechanisms and neurotoxicity pathways, employing advanced imaging technologies and molecular probe techniques to monitor the distribution, metabolism, and clearance processes of QDs in the nervous system in real time. A prominent contradiction in current research is that the ability of QDs to efficiently penetrate the BBB is both the foundation for their neural applications and a contributor to their neurotoxicity. Addressing this contradiction necessitates precise regulation of the size, surface charge, and targeting molecules of QDs to achieve controlled penetration. Additionally, it is crucial to emphasize research on the effects of long-term, low-dose exposure. Most current studies focus on acute toxicity effects, while the long-term retention of QDs in the CNS and their potential role in accelerating neurodegenerative diseases remain critical research directions in the field. Furthermore, from the perspective of environmental and sustainable applications, once released into the environment, QDs should be managed according to their elemental composition. For heavy metal-containing QDs (e.g., cadmium or lead), methods such as chemical precipitation, adsorption, or ion exchange can be used to immobilize and recover the heavy metal ions. For carbon- or silicon-based QDs, advanced oxidation processes (e.g., photocatalytic oxidation or Fenton reaction) can be employed to mineralize them into harmless small molecules. It is worth emphasizing that the recycling of QDs may also hold significant potential. Integrating circular economy principles into the life-cycle management of QDs will not only help reduce their environmental exposure risks but also promote the sustainable development of their clinical and industrial applications.

Looking ahead, advances in technologies such as single-particle tracking and in situ characterization will enable real-time, dynamic analysis of interactions at the quantum dot–neural interface. This will provide essential technical support for ultimately unraveling the complexities of quantum dot neurotoxicity.

## Figures and Tables

**Table 1:** Central Nervous System Toxicity.

No.	QDs Type	Model	Dose	Toxicity Mechanism	Toxicity Effects	Ref
1	CdTe/CdTe@ZnS	Mice, BV2 microglial cells	In vivo: 0.1 mL/20 g In vitro: 1.25, 5 nM	Induces ROS generation; activates the NLRP3 inflammasome	Neuroinflammation, hippocampal damage, cell death	[60]
2	CdSe/ZnS	Rats, Primary hippocampal neurons	In vivo: 20 nM In vitro: 10, 20 nM	Induces oxidative stress; triggers autophagy	impaired synaptic plasticity, memory deficits	[61]
3	CdSe	Primary hippocampal neurons	1–20 nM	Induces ROS; causes cytosolic Ca^2+^ overload	Neuronal dysfunction and death	[62]
4	CdSe	Primary hippocampal neurons	1–20 nM	Impaired sodium channel function; causes cytosolic Ca^2+^ overload	Neuronal dysfunction and death	[63]
5	CdSe/ZnS	GT1-7 neuronal cells	4 nM, 16 nM	Release of toxic metal ions (Cd^2+^, Zn^2+^) in lysosomes	Decreased cell viability and proliferation	[64]
6	CdTe	BV-2,HT-22 cells	0–10 μM	Cd^2+^ release; induces oxidative stress and DNA damage	Reduced cell viability; ultrastructural abnormalities in organelles	[65]
7	MPA-CdTe	Rats	0–1600 μg/mL	Suppresses ERK and PI3K-Akt signaling pathways	Impaired learning and memory; neuronal and synaptic structural damage	[66]
8	MPA-CdTe	BV-2 microglial cells	10–40 nM	Activates TLR2/MyD88/NF-κB and NLRP3 inflammasome pathways	Decreased cell viability and Microglial activation	[67]
9	MPA-CdTe	Primary hippocampal neurons	2.5–320 μg/mL	Oxidative stress; increased intracellular Ca^2+^	Neuronal apoptosis	[68]
10	CdTe/ZnS	Mice, BV2/HT22 cells	In vivo: 12.5 nmol/g In vitro 1.25 μM	Oxidative stress; activates mTOR signaling, inducing glycolytic shift	Altered microglial polarization; reduced neuronal count	[69]
11	CdTe/CdTe@ZnS	BV2 microglial cells	1.25–5 nM	Activates NF-κB and the NLRP3 inflammasome	Inflammatory cell death	[70]
12	MPA-CdTe	Rats	1600 μg/mL	Activates immune-related pathways (e.g., NOD/Toll-like receptor, NF-κB, TNF signaling)	Neuroinflammation; systemic immune response	[71]
13	CdTe/ZnS	Mice, BV-2 cells	In vivo: 0.1 mL/20 g In vitro: 1.25 μM	Disrupts glucose, lipid, and amino acid metabolism; induces inflammatory response	Metabolic disturbance; neuronal inflammatory injury	[72]
14	CdS	Rats, Neuronal cell line	In vivo: 0.1–25 mg/kg In vitro: 0.01–100 μg/mL	Induces oxidative stress and DNA oxidation damage	Neuronal degeneration, necrosis, and glial cell activation	[73]
15	CdTe-NALC	Mouse hippocampal neurons	0.01–1000 μmol/L	Oxidative stress; modulates the cAMP-CREB-BDNF signaling pathway	Suppressed neuronal activity; promoted apoptosis	[74]
16	CdSe/ZnS	Rats, Primary hippocampal neurons	0.5 nM, 10 nM	Oxidative stress	Impaired short-term plasticity and LTP; spatial memory deficits	[75]
17	CQDs	Mice	0.5, 5 mg/kg	Iron accumulation, lipid peroxidation, ferroptosis	Spatial learning/memory impairment, anxiety-like behavior, neuronal loss	[23]
18	CQDs	Mice	5 mg/kg	Oxidative stress; disruption of arginine/proline metabolism and unsaturated fatty acid biosynthesis	Metabolic dysfunction; organelle damage; cell death	[76]
19	MoS_2_	BV-2 microglial cells	0–200 μg/mL	Oxidative stress, lysosomal membrane permeabilization; NLRP3 inflammasome activation	Cytolysis; Inflammatory cell death	[77]
20	CsPbBr_3_	Human retinal organoids (hEROs)	25–100 μg/mL	Endoplasmic reticulum stress; upregulates apoptosis and downregulates retinal development pathways	Reduced retinal area/thickness; hindered retinal ganglion cell (RGC) differentiation	[78]
21	CsPbBr_3_	Mice, Neural stem cells (C17.2)	In vivo: 25 mg kg^−1^ In vitro: 0–100 μg/mL	induces oxidative stress and Ca^2+^ overload, activating caspase-3-mediated apoptosis	Mitochondrial dysfunction; hippocampal neuronal apoptosis	[79]
22	Ag_2_Se	Mice, BV2 microglial cells	In vivo: 15, 30 mg/kg In vitro: 2–8 μg/mL	Oxidative stress; activates NLRP3 inflammasome and Nrf2/PINK1-mediated mitophagy	Neuronal damage; microglial activation	[80]
23	MPA-CdTe	C57BL/6	Tail vein injection at 0.2 and 2 μg/g	Oxidative stress, neuroinflammation, and disruption of metabolic pathways	Motor behavior deficits, learning and memory impairment, and alterations in hippocampal cellular composition	[81]
24	Si QDs	SH-SY5Y	0, 0.025, 0.25, 2.5, 12.5, 25 μg/mL	Ferritinophagy, ferroptosis, and alterations in PD-related proteins	Decreased cell viability, mitochondrial dysfunction, and exacerbated injury in Parkinson’s disease models	[82]
25	N-GQDs A-GQDs	BV2 microglial cells	25 μg/mL, 100 μg/mL	Upregulation of circRNA: at low concentrations, inflammatory pathways; at high concentrations, calcium/GABA/olfactory pathways	Decreased cell viability and ultrastructural damage	[83]
26	GOQDs	Danio rerio	12.5, 25, 50, 100, 200 μg/mL	Accumulation of neurotoxic metabolites (3-HAA, QA) due to kynurenine pathway dysregulation	Adverse behavioral changes	[84]
27	CQDs	C57BL/6 mice, HMC3 cells, SH-SY5Y cells	In vivo: 8 mg/kg via tail vein injection; in vitro: 100 μg/mL	Microglia-mediated neuroinflammation and synergy between CQDs and MPP^+^	Motor dysfunction, brain tissue atrophy, dopaminergic neuron injury, and microglial activation	[85]
28	GQDs	NMRI mice	Intragastrically at 0, 10, 20, 40 mg/kg	Oxidative stress, mitochondrial dysfunction, apoptosis, and neuroinflammation	Decreased locomotor activity, increased anxiety-like behavior, memory impairment, and hippocampal neuron damage.	[86]

**Table 2:** Peripheral Nervous System Toxicity.

No.	QDs Type	Model	Dose	Toxicity Mechanism	Toxicity Effects	Ref
1	CdTe	Dorsal Root Ganglion (DRG) & Explants	10 μg/mL	Induces oxidative stress, lipid peroxidation, and release of Cd^2+^/Te^4+^ ions	Decreased cell viability, abnormal lipid droplet formation, impaired neurite outgrowth.	[87]
2	CdTe	Rat Schwann Cell Line (RSC96)	0–80 μM	Increases ROS, induces ER stress, and blocks autophagy flux	cell death	[88]
3	CdTe	ND7/23 Cell Line	1.25–40 μM,	Induces oxidative stress and mitochondrial apoptosis	Reduced cell viability, mitochondrial dysfunction.	[89]
4	CdTe	Rat DRG-derived ND7/23 Cells	10 μM	Activates ER stress and calcium signaling, leading to caspase-12 mediated apoptosis.	Ultrastructural damage to organelles, cell death	[90]

## Data Availability

No new data were created or analyzed in this study. Data sharing is not applicable to this article.

## Abbreviations

The following abbreviations are used in this manuscript:

BBB	blood–brain barrier
CQDs	carbon quantum dots
CNS	Central Nervous System
LA	lipoic acid
LTP	long-term potentiation
mtROS	Mitochondria-derived ROS
NPs	nanoparticles
NAC	N-acetylcysteine
PNS	Peripheral Nervous System
PEC	predicted environmental concentrations
PINK1	PTEN-induced kinase 1
QLED	Quantum Dot Light Emitting Diodes
QDs	Quantum Dots
ROS	reactive oxygen species
NCX	reverse sodium-calcium exchanger
Nrf2	the nuclear factor erythroid 2-related factor 2
VGSCs	voltage-gated sodium channels
α-syn	α-synuclein
SiQDs	Silicon quantum dots
PDT	photodynamic therapy
PTT	photothermal therapies
